# RELATING SPECIFIC MEDICATIONS TO RECORD OF FALLS IN A LARGE COHORT OF OLDER SWEDISH ADULTS

**DOI:** 10.1093/geroni/igae098.4065

**Published:** 2024-12-31

**Authors:** Daniel Peterson, Linda Johansson, Bjorn Westerlind, Thais Lopes De Oliveira, Deborah Finkel

**Affiliations:** Arizona State University, Arizona State University, Arizona, United States; School of Health and Welfare, Jönköping University, Jönköping, Jonkopings Lan, Sweden; Jonkoping University, Jönköping, Jonkopings Lan, Sweden; Karolinska Institutet, Stockholm, Stockholms Lan, Sweden; University of Southern California, Los Angeles, California, United States

## Abstract

Falls are common and devastating for older adults and can be impacted by medication. However, analyses relating medication and falls often focus on a subset of medications. We assessed relationships between medication prescription and prospectively captured falls within a large, Swedish National Quality Register (Senior Alert) sample. Participants (N=441) were tracked for approximately 2.5 years. The observation period was separated into 30-day windows, within which the presence of medication prescription and number of falls were noted. To relate medication prescription and falls, we used Logistic Generalized Estimating Equation (GEE) self-comparison models, considering the dependence of longitudinal data as a cluster to correct confidence intervals and individuals as the cluster. Total number of medications for each 30-day window was included in the model. 286 unique medications were identified and initially considered. However, for model convergence, only medications that were prescribed 100 or more times across all participants and timeframes were included in the analyses, leaving 86 medications. After correction for multiple comparisons, 3 classes of medications were related to falls (annotated with 5-digit ATC codes): B01AB (antithrombotics), B03BB (folic Acid), and A03AX (functional gastro-intestinal [GI] disorder medications). Findings are partially consistent with previous work, as antithrombotic agents were related to falls. Folic acid and GI medications were not previously related to increased fall-risk. Results extend previous work in several ways. 1) data were analyzed via a robust, within subject design, 2) data were collected in older adults living in a novel, Swedish cohort, and 3) we interrogated many common medications.

